# Dynamic denominators: the impact of seasonally varying population numbers on disease incidence estimates

**DOI:** 10.1186/s12963-016-0106-0

**Published:** 2016-10-12

**Authors:** Elisabeth zu Erbach-Schoenberg, Victor A. Alegana, Alessandro Sorichetta, Catherine Linard, Christoper Lourenço, Nick W. Ruktanonchai, Bonita Graupe, Tomas J. Bird, Carla Pezzulo, Amy Wesolowski, Andrew J. Tatem

**Affiliations:** 1 WorldPop, Geography and Environment, University of Southampton, University Road, Southampton, SO17 1BJ UK; 2 Flowminder Foundation, Roslagsgatan 17, 113 55 Stockholm, Sweden; 3 Spatial Epidemiology Lab (SpELL), Université Libre de Bruxelles, Av. FD Roosevelt 50, 1050 Brussels, Belgium; 4 Department of Geography, Université de Namur, Rue de Bruxelles 61, 5000 Namur, Belgium; 5 Clinton Health Access Initiative, Boston, MA USA; 6 Mobile Telecommunications Limited, Windhoek, Namibia; 7 Center for Communicable Disease Dynamics and Department of Epidemiology, Harvard, Boston, MA USA; 8 Department of Ecology and Evolutionary Biology, Princeton University, Princeton, NJ USA; 9 Fogarty International Center, National Institutes of Health, Bethesda, MD 20892 USA

**Keywords:** Health metrics, Mobile phones, Malaria, Surveillance, Disease incidence, Seasonality

## Abstract

**Background:**

Reliable health metrics are crucial for accurately assessing disease burden and planning interventions. Many health indicators are measured through passive surveillance systems and are reliant on accurate estimates of denominators to transform case counts into incidence measures. These denominator estimates generally come from national censuses and use large area growth rates to estimate annual changes. Typically, they do not account for any seasonal fluctuations and thus assume a static denominator population. Many recent studies have highlighted the dynamic nature of human populations through quantitative analyses of mobile phone call data records and a range of other sources, emphasizing seasonal changes. In this study, we use mobile phone data to capture patterns of short-term human population movement and to map dynamism in population densities.

**Methods:**

We show how mobile phone data can be used to measure seasonal changes in health district population numbers, which are used as denominators for calculating district-level disease incidence. Using the example of malaria case reporting in Namibia we use 3.5 years of phone data to investigate the spatial and temporal effects of fluctuations in denominators caused by seasonal mobility on malaria incidence estimates.

**Results:**

We show that even in a sparsely populated country with large distances between population centers, such as Namibia, populations are highly dynamic throughout the year. We highlight how seasonal mobility affects malaria incidence estimates, leading to differences of up to 30 % compared to estimates created using static population maps. These differences exhibit clear spatial patterns, with likely overestimation of incidence in the high-prevalence zones in the north of Namibia and underestimation in lower-risk areas when compared to using static populations.

**Conclusion:**

The results here highlight how health metrics that rely on static estimates of denominators from censuses may differ substantially once mobility and seasonal variations are taken into account. With respect to the setting of malaria in Namibia, the results indicate that Namibia may actually be closer to malaria elimination than previously thought. More broadly, the results highlight how dynamic populations are. In addition to affecting incidence estimates, these changes in population density will also have an impact on allocation of medical resources. Awareness of seasonal movements has the potential to improve the impact of interventions, such as vaccination campaigns or distributions of commodities like bed nets.

**Electronic supplementary material:**

The online version of this article (doi:10.1186/s12963-016-0106-0) contains supplementary material, which is available to authorized users.

## Background

The Sustainable Development Goals (SDGs) aim at a significant reduction in the burden caused by communicable diseases, most prominently AIDS, malaria and tuberculosis [1]. Accurate measurements of disease incidence are key for monitoring progress towards these goals and for targeting resource allocation and intervention activities to further reduce disease burden [2]. Many SDG health indicators such as disease morbidity and mortality are measured through passive surveillance systems reporting at the level of health facilities or districts and are reliant on estimates of facility catchment or district populations to convert case counts to population-level metrics. Incidence-reporting, the number of reported cases divided by the population size (denominator), is used in many large international efforts, ranging from the assessment of the global burden of disease, such as malaria or tuberculosis [3–5], to routine government surveillance to guide resource allocation, interventions and elimination efforts. Improving surveillance, diagnostics and measurement methods has received substantial focus recently, aiming to improve quality and coverage of case data as well as rapidity of reporting [6–9]. However, reliable and contemporary case records are only part of the equation and the task of improving population denominator estimates has received much less attention [10].

Accurate data on the distribution, and ideally demographics, of the population is crucial for reliable incidence estimates at subnational scales. Where contemporary denominator data are not available, reporting case numbers instead of incidence is generally the only option. This leads to a bias in reported disease burden as more populated areas will naturally have more cases. Typically, denominators are based on static census-derived estimates or annual projections from these baselines, but this approach has two main limitations. First, in many low-income settings, census population counts can be unreliable and outdated [10]. Methods based on satellite imagery and aerial photography continue to be explored for estimating population counts and distributions in the absence of census numbers [11, 12], but these, like a census, only provide a single snapshot of estimates, often missing substantial seasonal changes in population distributions.

Many studies have highlighted the dynamic nature of human populations through quantitative analyses, particularly recent studies in low income settings [13–16]. Movements span multiple timescales and are driven by a variety of factors: from long term migration and crisis-induced displacements, to short term seasonal movements [17–23]. Seasonal movements can be observed in all countries [13, 15, 24–28], with holidays, school terms and agricultural seasons being key drivers. These strong seasonal movements lead to changes in population distributions, which result in changing denominators that cannot be captured through simple projections from census counts. Nevertheless, assessments of disease burden, calculations of health facility budgets, staffing and stocks, and routine intervention delivery are all planned based on static denominators. In the past, methods for capturing seasonal movements and the resulting changing population distributions have been unavailable, since information over large spatial extents and high temporal resolution are needed to capture these movements. Satellite nightlights have been shown to be a useful source for capturing seasonal migration patterns in low income regions [13], but these data only capture relative changes in brightness at the edges of large cities, produce no information for rural areas and only provide approximate estimates on the timings of substantial migration events. Travel history surveys can provide valuable information, but are limited to small areas and sample sizes and also suffer from recall bias.

Novel sources of data on human movements that may be capable of capturing seasonal movement patterns with high temporal resolution and over large spatial extents have recently become available [17]. With high mobile phone ownership and usage rates, even in low-income settings [29], large volumes of data on population movements at unprecedented spatial and temporal resolution are obtainable using inferred location data from mobile phone calling records. These *call data records* (CDRs) are recorded by mobile phone operators for billing purposes and include the location of the mobile phone tower through which calls and text messages are routed. Changes in the tower that an individual’s communications are routed through can be used to measure individual movements, which can then be aggregated to produce flow estimates across differing spatial and temporal scales [30, 31]. Such data are being recorded continuously and have been used in several contexts to assess human movements and changes in population distributions. Notable example applications are quantifying the impact of mobility on malaria risk [22, 24, 25, 32] and other diseases [23, 33], as well as measuring displacements after natural disasters [30, 34, 35]. Other recent analyses have also shown the potential of using CDRs to produce accurate and seasonally varying population distribution maps [28].

In this paper, we demonstrate how CDRs can be used to estimate changing population distributions subnationally. Using the example of *P.falciparum* malaria in Namibia, which is aiming for elimination of the disease, we show how estimates of seasonally changing health district denominators result in changing incidence estimates over the static denominators used at present to derive malaria incidence estimates. We show that taking into account seasonal fluctuations of population density affects incidence estimates and highlight potential areas of overestimated and underestimated incidence.

## Methods

### Census

**Fig. 1:**
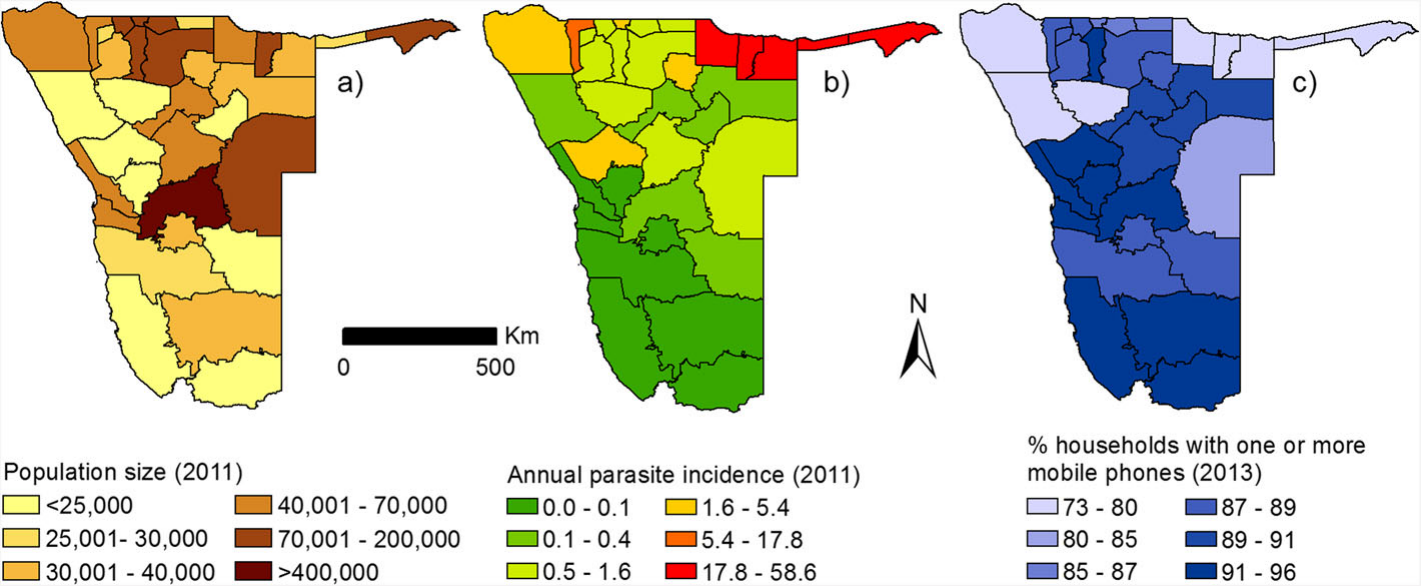
Population size, malaria incidence, and mobile phone ownership in Namibia: **a** Population numbers per health district according to 2011 census, **b** Annual parasite incidence 2011 using census population numbers as denominator, **c** mobile phone ownership according to DHS 2013

We obtained data on population counts from the most recent Namibia census, conducted in September 2011 [36]. To obtain census counts at the health district level, we summed the population counts for all administrative units contained within a health district. The resulting data are shown in Fig. 1a. As censuses are typically undertaken every 10 years, population projections are generally used as denominators in disease incidence calculations and we therefore use population projections provided by the Namibian Statistics Agency (NSA) [37] for quantifying incidence in 2012–2014. Since these projections are on region level, we calculated the predicted rate of increase for each region and then assigned this rate to all health districts contained within that region to obtain projections for each health district for each year. Here we will refer to these projected population numbers as the ‘*static denominators*’*.*

### Malaria data

**Fig. 2:**
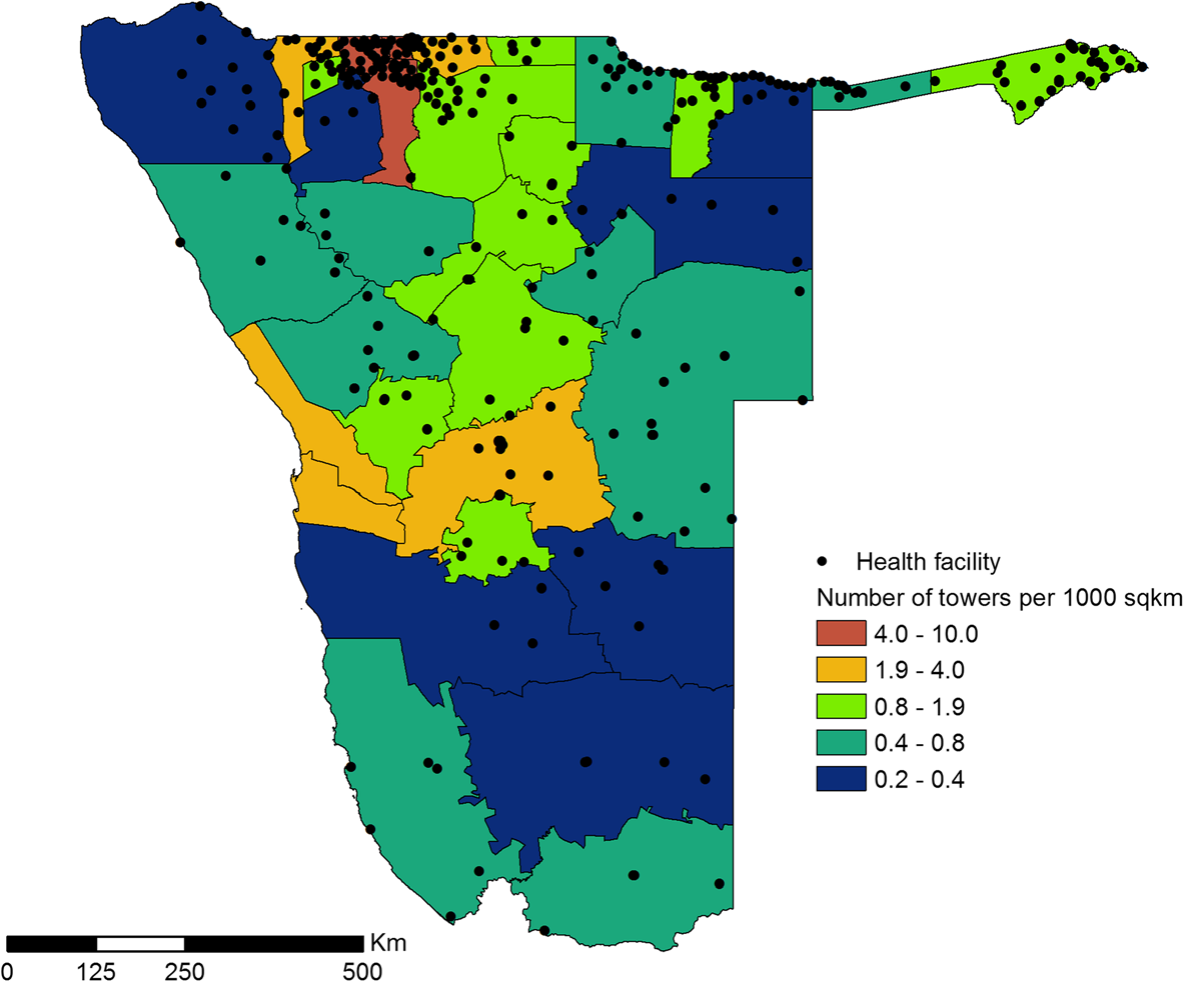
Health facility locations and mobile phone tower density: Health facility locations for facilities with completed case reports. Colour of health districts according to tower density as towers per 1000 km^2^

Malaria case data from both public and private health facilities in Namibia (*n* = 356 reported malaria case data, Fig. 2) from January 2010 to May 2014 were obtained from the Namibia National Vector-borne Diseases Control Programme (NVDCP). There were 469 facilities in total in Namibia between 2010 and 2014, of which 377 (80.3 %) are managed in the public sector (Ministry of Health and Social Services, missions, non-governmental organisations and Ministry of Defence and police) while 92 (19.7 %) belong to the private sector managed by private individuals. Malaria case data represented confirmed *P.falciparum* malaria cases for the study period for all ages. The number of cases varied by year and were lowest in 2012 (*n* = *3299*) and highest in 2010 (*n* = *26,373*). For the majority of primary facilities, Rapid Diagnostic Tests (RDTs) were used routinely to examine blood samples from most patients although a few were examined using microscopy [38], mostly at secondary and tertiary facilities. Since it was not possible to distinguish cases that had been confirmed using an RDT or via microscopy, there was no stratification based on diagnosis. In total, the data were generally complete (over 90 %), in terms of reporting rates for the majority of facilities, with zero recorded cases referring to no confirmed malaria cases. Case counts were aggregated by month and health district for the purposes of the analyses undertaken here. Figure 1b shows the annual incidence (sum of all cases over the year) for 2011 using census population counts as the denominator. Spatial differences in incidence are evident, with high incidence in the north-east and low incidence in the south.

### Mobile phone call data records (CDRs)

**Fig. 3:**
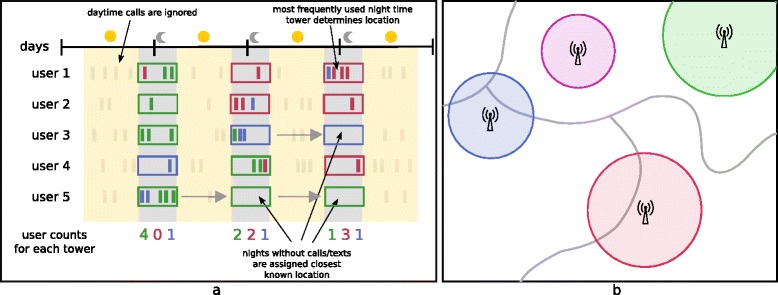
CDR data processing method illustration: **a** Extracting unique users per tower from raw CDR data. **b** Redistribution of user counts from tower level to health district level based on areas of intersection

Mobile phone operators routinely collect CDRs for billing purposes. CDRs typically include the date and time of all communications (including SMS and calling), an anonymised identifier code for the user who made or received the communication and the tower through which the call or text was routed. From the CDRs, daily locations of users can be calculated by determining the most frequently used tower for each individual and day. Previous studies have shown that using night time CDRs leads to more accurate population density estimates when compared against census-derived counts [28]. Thus, to ensure comparability with the census-derived counts [36], only night-time communications were used. We determined night-time as the time between 8 pm and 6 am (the following morning), with calls made between midnight and 6 am being counted towards the previous day (see Fig. 3a). Once we determined individual’s locations for each day, any days without night time communications (and thus undefined location) were assigned the closest known location, either backwards or forwards in time. This reduced stochastic fluctuations in the data resulting from varying usage rates by providing a stable underlying user population.

For the example case of Namibia discussed here, we used a data set spanning 43 months from October 2010 to May 2014. The data set contains 72 billion communications and was provided by the leading mobile phone provider in Namibia, MTC. This data set covers a high proportion of the population, as Namibia has relatively high phone ownership rates, even in rural areas (Fig. 1c) [39], MTC has a very high market share of the Namibian mobile communications market (76 % for 2010–2012) [40, 41] and all health districts are covered by mobile phone towers (Fig. 2).

The resulting data set from Namibia contained the number of unique users per day for each tower. To aggregate to the coarser scale of months to align with the malaria case data, we calculated the mean daily number of users for each tower. To obtain health district level user numbers from this, we followed the methods described in Deville et al. [28], allocating numbers of users to health districts based on the area of intersection between tower reception areas and health districts. If the area covered by a certain tower was entirely within one health district, all population associated with that tower were counted towards the total of the health district. If the coverage area of a tower spanned two or more health districts, the number of users was divided across the health districts based on the area of overlap between the coverage area and the respective health district (see Fig. 3b). Tower coverage is generally approximated by Voronoi polygons if no other information is available, but for the setting of Namibia, MTC provided approximate ranges of their towers which we used instead to more accurately determine coverage areas of towers and intersections with health district.

The method discussed here, provided us with monthly user numbers for each health district which we used to assess denominator changes.

### Denominator changes

For a given month and health district, we calculated a ratio representing relative density of users in the health district during that month, compared to density of users during the census period (September 2011). While the 28 August 2011 is listed as the official census date, in some remote areas the enumeration was reported to have taken up to mid-September [42], therefore we used September 2011 as the census month.

Rapid changes in user numbers were assumed to be the result of increases or decreases in population numbers. Thus, change in user numbers was used to estimate the changes in population distribution, and the ratio of change derived from the CDRs was applied to the census population count of each health district. We then adjusted those estimates to match the projected population totals [37]. We use these estimates as the ‘*dynamic denominators*’ for comparison against the static denominators*.*

The adjustment to match total population numbers is necessary since the data set spans a long period of time and an increase in user numbers over this period was observed (Additional file 1: Figure S2). Since mobile phone penetration rates are still far from 100 % in most low and middle income countries (especially in rural areas), we expect to see an increase in mobile phone ownership over time and therefore increasing number of users. Additionally, given the length of the time period covered by the CDR data set, there will have been an increase in actual population numbers as well. We therefore use the projected population numbers to adjust our estimates, as we expect growth of the user base to be faster than the population growth. Additionally, this enabled comparison between incidence estimates using static and dynamic denominators.

## Results

**Fig. 4:**
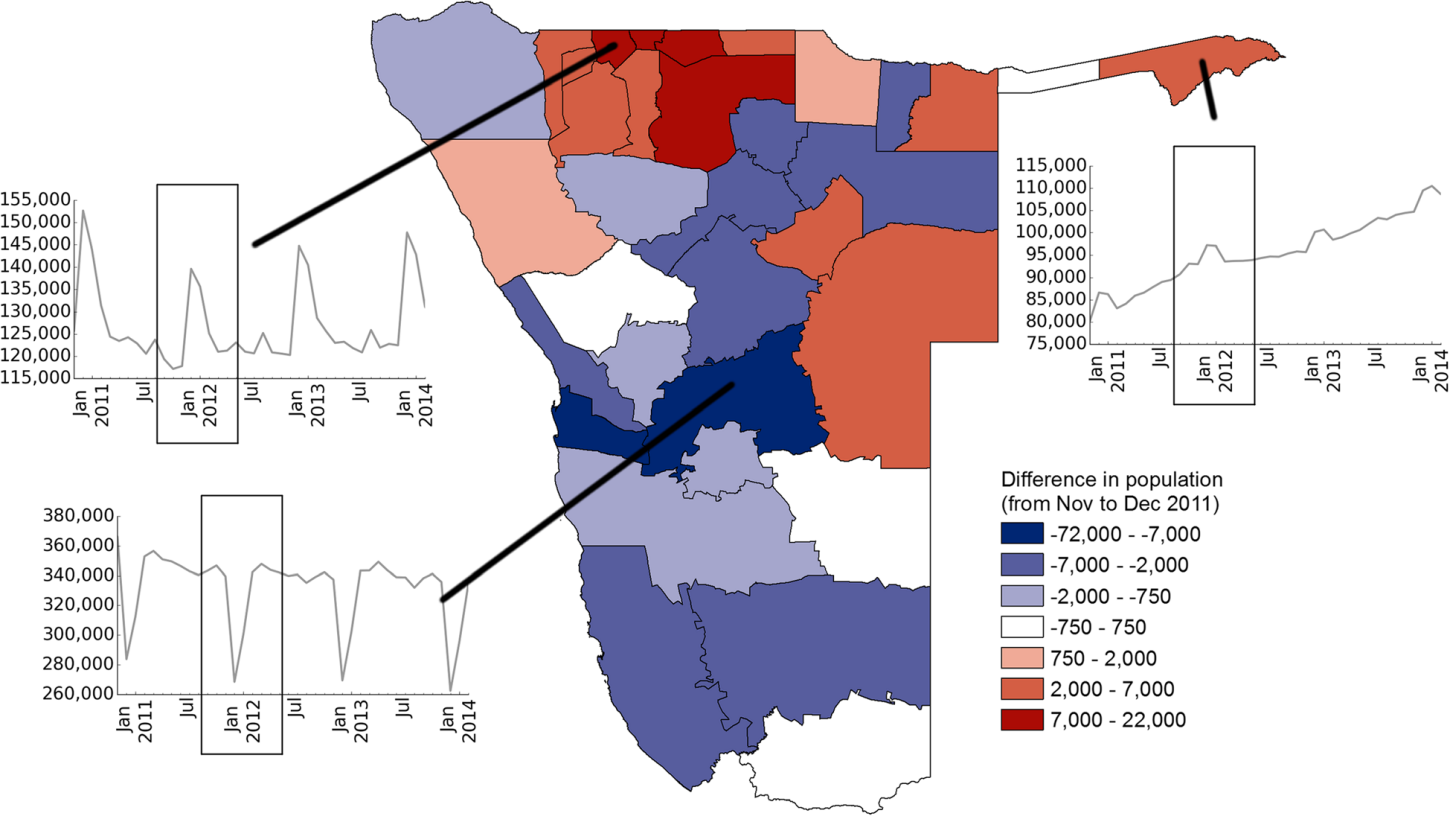
Seasonal changes in population numbers: Difference in predicted population number between November and December 2011 for each health district. Insets show predicted population number for selected health districts over the whole study period

Compared to the use of non-projected census counts, using projected estimates accounts for estimated population growth. However, projected estimates still fail to account for seasonal changes. The dynamic denominators used here capture and quantify the intra-annual changes in the population distribution over time (Fig. 4). These changes in the population distribution are the result of population movements, measured here as movements between health districts. The majority of these movements are seasonal and occur around holiday periods, with the most prominent change happening around Christmas time. In December, substantial population movements from the capital, Windhoek, to the north of the country are evident, most likely caused by people visiting friends and relatives (Fig. 4). This movement is reversed in January with people returning home. Relatively smaller, but still significant movements like this can be seen later in the year, for example around Easter. The change in population distribution around Christmas is of particular importance, due to the magnitude of population flows as well as that time coinciding with the early part of the malaria transmission season. Note that while clear seasonal patterns exist, variation between years is also evident in the magnitude and timing of peaks (Fig. 4).

**Fig. 5:**
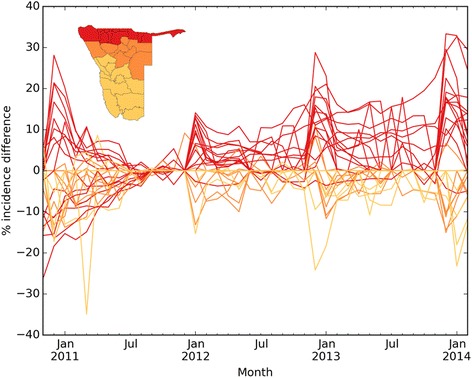
Difference in incidence estimates using dynamic and static denominators: Difference between dynamic and static incidence as percent of dynamic incidence estimate. Colour of lines according to malaria risk zone classification of the corresponding health district as shown in inset map

**Fig. 6:**
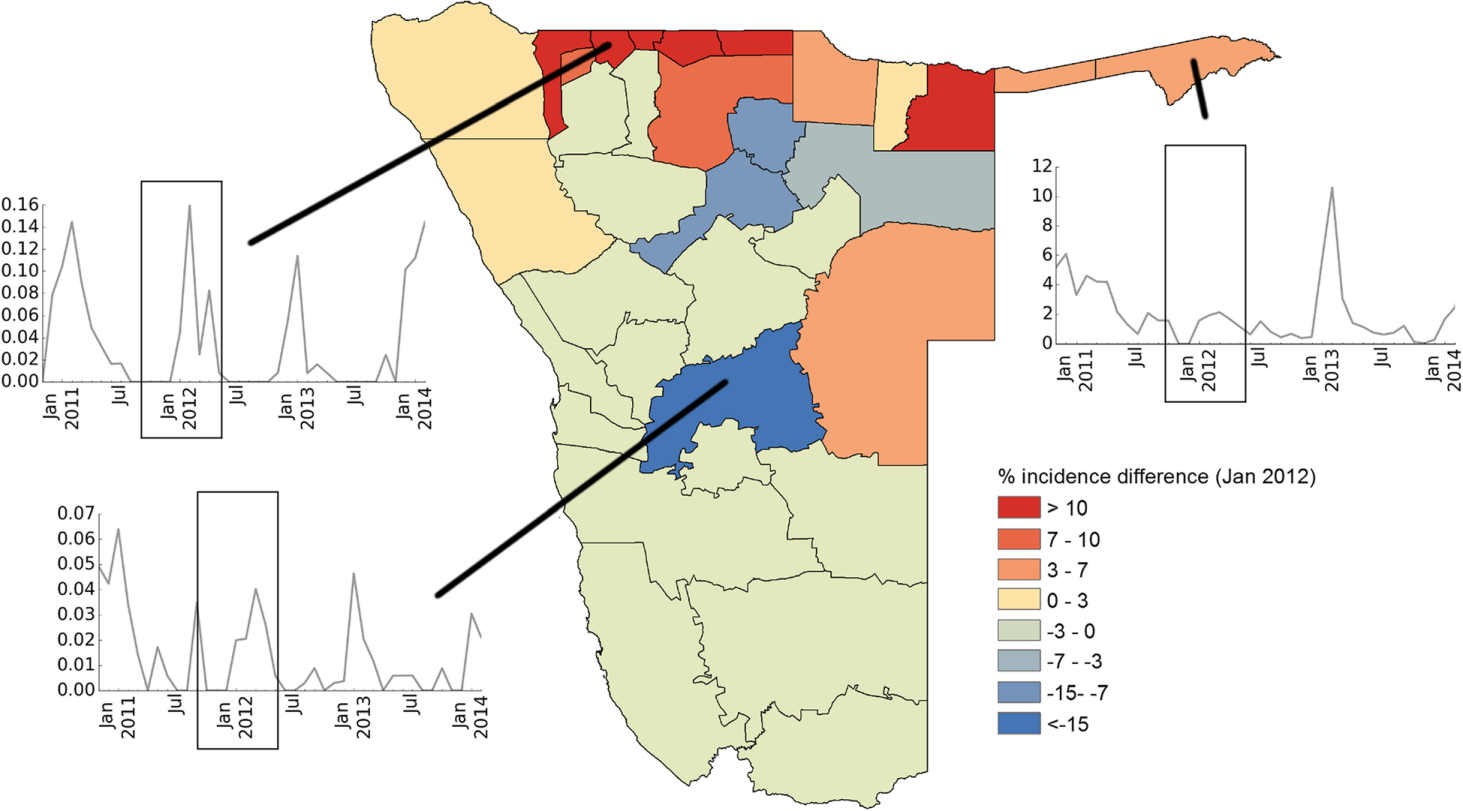
Difference between dynamic and static incidence for January 2012: Difference between dynamic and static incidence as percent of dynamic incidence estimate for each health district for January 2012. *Red* indicating overestimation of incidence using the static denominator and *blue* corresponding to potential underestimation. Insets show the dynamic incidence for selected health districts over the whole study period

To assess the impact of these changes in population distribution on disease incidence estimates, we calculated the monthly *P.falciparum* malaria incidence for the 2011–2014 period using both the static and dynamic denominators, to create ’static’ and ‘dynamic’ incidence estimates for each health district and quantify their differences. Figure 5 shows the difference between the static and dynamic incidence estimates as percentage of the dynamic estimate, with separate lines for each health district. We coloured the lines according to the NVDCP health district classification into three malaria risk zones which range from 1 (high risk, red) to 3 (low risk, yellow), (see Fig. 5, inset map). This figure shows that compared to the dynamic incidence estimates (which take into account seasonal fluctuations in population distribution), the static estimates are likely overestimating actual incidence by up to 30 % for the northern higher risk zone, especially for the beginning of the peak malaria transmission season in December/January. In the zones of lower risk (zones 2 and 3, orange and yellow), using static denominators underestimates incidence by up to 30 %. Figure 6 shows the difference between incidence estimates using static and dynamic denominators for January 2012. The high risk zones in the north of Namibia mostly exhibit overestimation of incidence when using static denominators, while areas with lower incidence show underestimation (such as Windhoek in the center with more than 10 % underestimation). Incidence changes over time are shown for several select health districts, highlighting the seasonal transmission of malaria in Namibia (Fig. 6).

## Discussion

Accurate and recent subnational data on population sizes and distributions in low- and middle-income countries are valuable for constructing health and wealth related metrics as well as improving geographically targeted policies for reducing inequalities among and within countries [43, 44]. In the context of the SDG health metrics, which aim at targeting the most vulnerable populations, reliable datasets on the distribution of the population at subnational scales provide a solid base for accurately identifying individuals at risk of contracting diseases [44], and for monitoring progress in reducing disease burden over time and space [45].

The results shown in this paper highlight that seasonal mobility and resulting changes in population distribution can affect subnational incidence estimates substantially, which in turn impact disease burden and distribution estimates. Namibia’s current malaria strategy aims to achieve a national case incidence of less than 1.0 per 1000 population by 2016 [38], and mapping incidence subnationally provides important indicators of progress towards this aim as well as measuring seasonal changes and highlighting key regions for targeting. The likely overestimation of incidence in the high-risk areas in the north of Namibia at certain times of year when using static denominators (Fig. 5) implies that Namibia may well be closer to elimination than previously thought. At the same time, consistent underestimation in the lower-risk zones as a result of seasonally changing population numbers could lead to insufficient allocation of resources to keep areas of unstable transmission malaria free.

In addition to being crucial as denominators to assess disease burden, contemporary and reliable population data are needed for planning and resource allocation. Disease prevalence and population distributions change with time, especially in the decade or longer between censuses. On shorter time scales, seasonal mobility leads to variations in health facility service and stock demand. Without information on population dynamics, staffing and resource allocation, decisions have to be made using static and potentially outdated catchment population numbers. Where mobility in low income regions has been explored at national scales, strong seasonal patterns are evident (e.g. [13, 15]), leading to increased pressure on health services in certain regions, depending equally on interactions with seasonally varying pathogen dynamics. In addition to estimating incidence, updated population counts can be used to assess seasonally varying demand on health systems, thus providing a broader scope for these data than communicable diseases. Preparedness for variation in demand on health facilities (especially seasonal increases in demand) can ensure more reliable service provision from communicable diseases, to non-communicable and chronic diseases. Seasonally variable population distribution maps are also important for survey design, where knowledge of seasonal fluctuations is important for defining population sizes and capturing the demographics of groups that engage in seasonal migration.

While the methods presented here facilitate assessment of seasonal changes in population distribution and the resulting impact on incidence estimates, limitations do exist. Since the method relies on the census population counts for transforming changes in phone user numbers into changes in population numbers, any inaccuracies in the census numbers will affect downstream estimates. Therefore, this approach cannot be used to assess the accuracy of a census or improve on it other than to provide more up to date estimates following seasonal changes. Another issue is data coverage, and while 95 % of the population of Namibia lives in areas with mobile phone coverage [46], there are large areas (mainly desert) without coverage. Populations living in or temporarily moving into areas without phone coverage cannot be accounted for by the methods outlined here, which will be problematic for countries with lower coverage, though this is a declining problem as mobile phone coverage continues to rise globally (http://www.gsma.com/mobileeconomy/global/2015/GSMA_Global_Mobile_Economy_Report_2015.pdf). Similar issues arise in settings with low mobile phone ownership rates, which tend to be biased towards the least accessible and poorest population groups [47], though again, these biases are decreasing as phone ownership rises (http://www.gsma.com/mobileeconomy/global/2015/GSMA_Global_Mobile_Economy_Report_2015.pdf). Household surveys could help assess which parts of the population are potentially under-represented by CDRs. Depending on the survey, they can provide information on phone usage and ownership patterns and allow assessment of spatial differences that could bias results. For Namibia, we have used the DHS from 2013 [39] to assess geographical differences in household phone ownership.

The aim here was simply to improve estimates of catchment facility denominator dynamics over existing census-based numbers to refine disease incidence metrics. It is clear, however, that information on treatment seeking behaviours would further improve the value of the outputs. People travelling may seek treatment away from home or prefer to seek treatment at their place of residence. Without explicit data on treatment seeking rates, we cannot further refine the relationship between population distribution and health facility catchment sizes. The incubation period of malaria adds another area of uncertainty in the dynamic incidence measures presented here, as for some cases the appropriate denominators may actually be from the previous month. While the focus of the example presented here is malaria, the same method can be applied to other diseases. However, it is important to take into account the time scales considered. For diseases with longer incubation periods, such as TB or HIV, long term migration data from censuses or travel surveys may be a suitable source for understanding dynamics. However, where migration data from censuses or surveys are unreliable or outdated, CDRs also can be used to assess population movements over longer temporal scales.

Building on this work of defining changes in population size over time, ongoing research is focussed on the mapping of absolute population numbers directly from CDRs, rather than relative changes, through adaptation of previously developed models [28]. This research will likely require adaption to the context of to low and middle income countries that typically have incomplete network coverage and lower phone ownership, using spatial modelling techniques [48] to improve spatial accuracies where network coverage is poor. Additionally, integrating survey data on phone ownership and usage will aid in addressing demographic and cultural biases.

CDRs are collected continuously by mobile phone providers, but due to privacy concerns access is strictly regulated and thus restricted. Issues with anonymity have been raised, specifically for individual level mobility data [49]. However, the data required for applying the methods presented in this paper are far less sensitive, as the approach relies solely on user counts for given spatial units, thus not containing any individual level information or movement information. This could open up the possibility of ongoing, near-real time data feeds, which would allow for such data to be dynamically integrated into health information systems through collaboration between network operators and governments. Supported by appropriate incentives, this would improve incidence-based metrics, allow better assessment health system demands as well as demands on services in general.

## Conclusion

The advent of the SDGs, as well as increasing global focus on disease elimination and health metrics, is producing a greater emphasis on improving disease case detection for surveillance at fine spatial scales. However, in most cases the denominator data used to then construct incidence estimates come from aging and static census data. Here, we have demonstrated that seasonal movements lead to changes in denominators, which in turn affect incidence estimates. In the example of malaria in Namibia, the results indicate that Namibia may actually be closer to malaria elimination than previously measured using denominator data that do not account for seasonal movements. We have shown how these movements that lead to changing denominators can be measured using mobile phone CDRs. Accurately measuring changes in population distribution can be crucial for monitoring communicable and vector-borne disease dynamics as well as intervention planning and resource allocation.

## Additional file

Additional file 1: S1-Supplemental figures. (PDF 1136 kb)

## Abbreviations

CDR: Call data records
SDG: Sustainable development goals
