# Supplementary material for: Dynamic denominators: the impact of seasonally varying population numbers on disease incidence estimates
Source: Popul Health Metr. 2016 Oct 12;14:35. doi: 10.1186/s12963-016-0106-0 (PMC5062910; doi:10.1186/s12963-016-0106-0)
Supplement: Additional file 1: — S1-Supplemental figures. (PDF 1136 kb) [file 12963_2016_106_MOESM1_ESM.pdf]

Supplemental to **Dynamic denominators: The impact of seasonally-varying population numbers on disease incidence estimates**

1 Study area

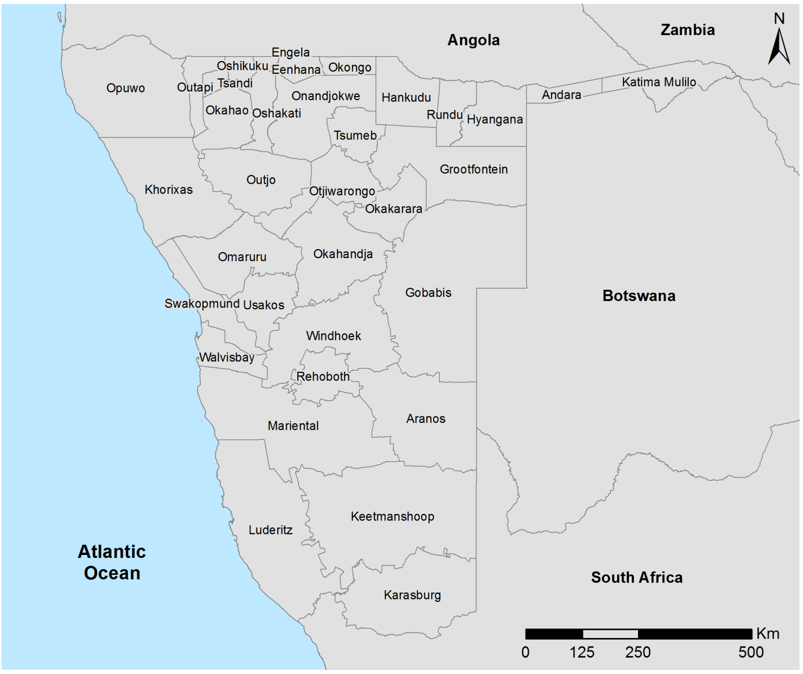

Figure 1: Study area and health district names.

2 CDR data additional information

Figure 2 shows the total number of unique users over the whole study period. To determine a user's location only nighttime calls (8pm-6am) are used. On days without any communications, an individual's location is undefined. To create a stable user population over time, we fill in the missing location with the closest known location (either backward or forward in time) in this case. A user's locations are filled in between the first and last day for which the indidvidual has a defined location, but not beyond that. This is why we see an increase in users at the beginning of the study period and a decrease at the end, since users who call or text infrequently (i.e., not every day) are likely to register for the first time several days after the beginning of the data set. Therefore we restrict the analyses to the period of November 2010 - February 2014 (shown as light grey lines) to exclude the initial and final period during which user numbers change rapidly, which is why in the main manuscript data is shown only for this period. Note that the CDR data was provided in two separate datasets, one ranging from October 2010 to November 2011 and the second from November 2011 to April 2014. November 2011 is marked as a black line. The rate of increase

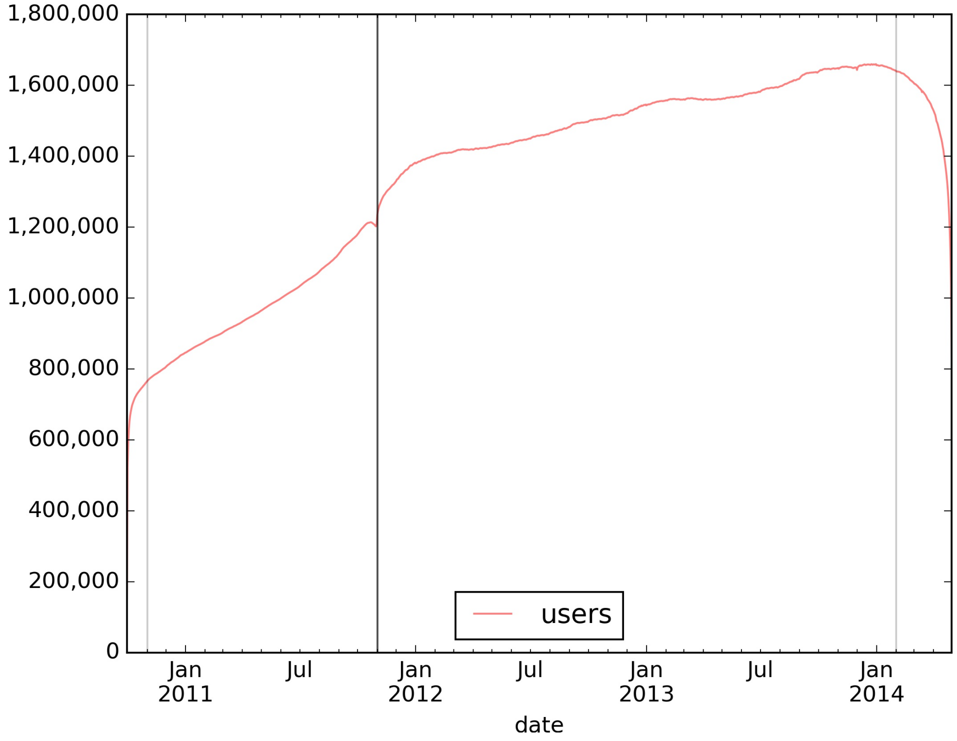

Figure 2: Number of users over time.

in user numbers varies slightly between the two data sets due to a more restrictive extraction procedure used for the first data set. However, since predicted population numbers are scaled to match projected population totals this should not impact the final predictions.

3 Results - additional figures

3.1 Malaria risk zone level

Figure 3 shows the monthly predicted total population numbers for each of the three malaria risk zones (solid lines). Census numbers (constant, dashed line) and yearly projections (markers with connected by dotted line) are shown for comparison. Compared to the projections, we observe a larger increase in the population predictions for the high risk zone (1) in the north of the country and a relatively lower increase in zone 3 in the south, which includes the capital, Windhoek. This could be a result of biases in the data such as a larger increase in user numbers in the north of the country. However, preliminary analyses indicate that this is not the case. Figure 4 shows the number of users for each malaria zone and highlights the fact that zone 1 does not have a larger increase in user numbers than zone 3. Therefore, it is unlikely that the observed difference between population estimates and projections are a result of different mobile phone uptake rates. The observed differences to the projections are potentially a result of actual internal redistribution of population, although this needs further investigation.

Figure 5 shows the difference between static and dynamic incidence as percentage of the commonly used dynamic incidence. This figure displays the same information as Figure 5 in the main manuscript, but aggregates all health districts belonging to the same malaria risk zone.

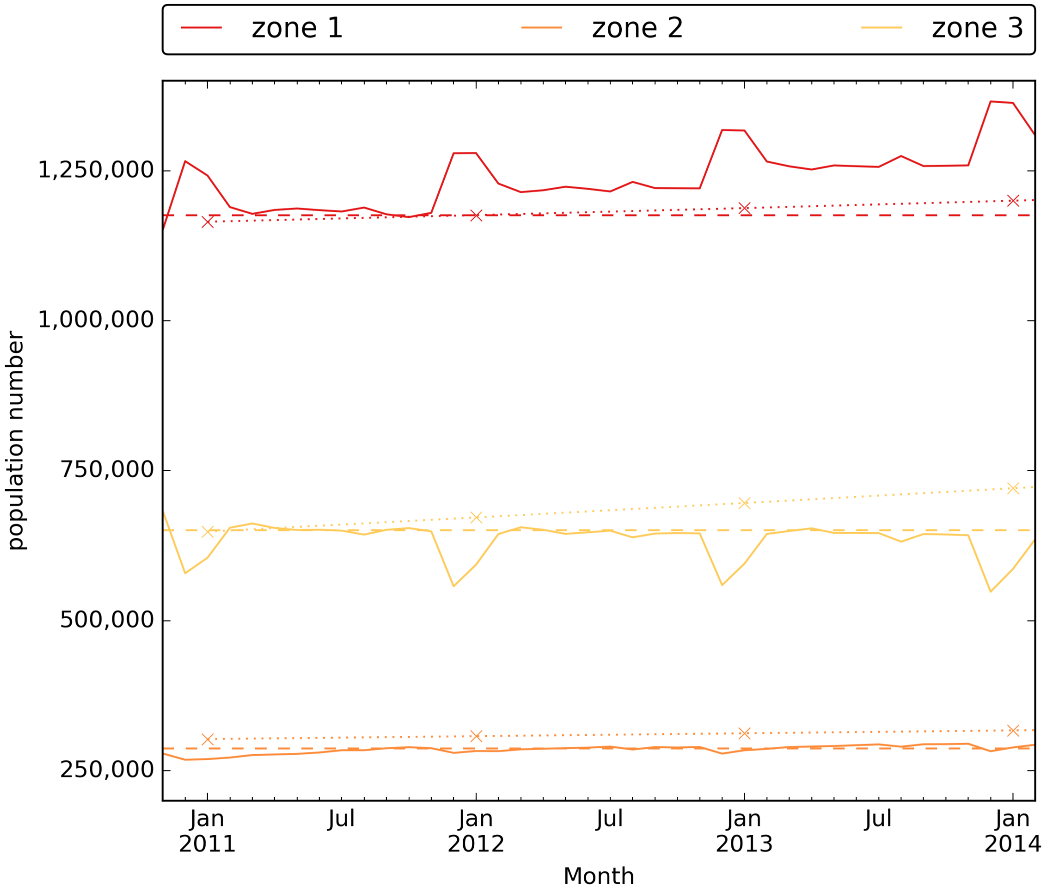

Figure 3: Population predictions, projections and census numbers.

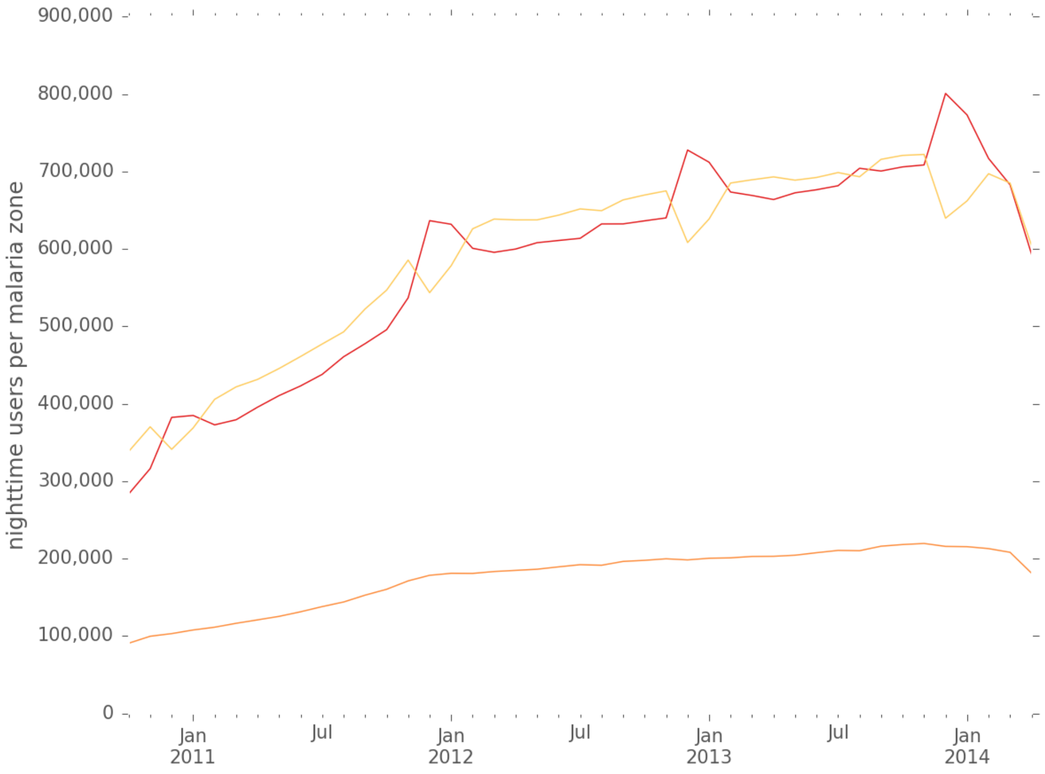

Figure 4: Number of users in each malaria zone.

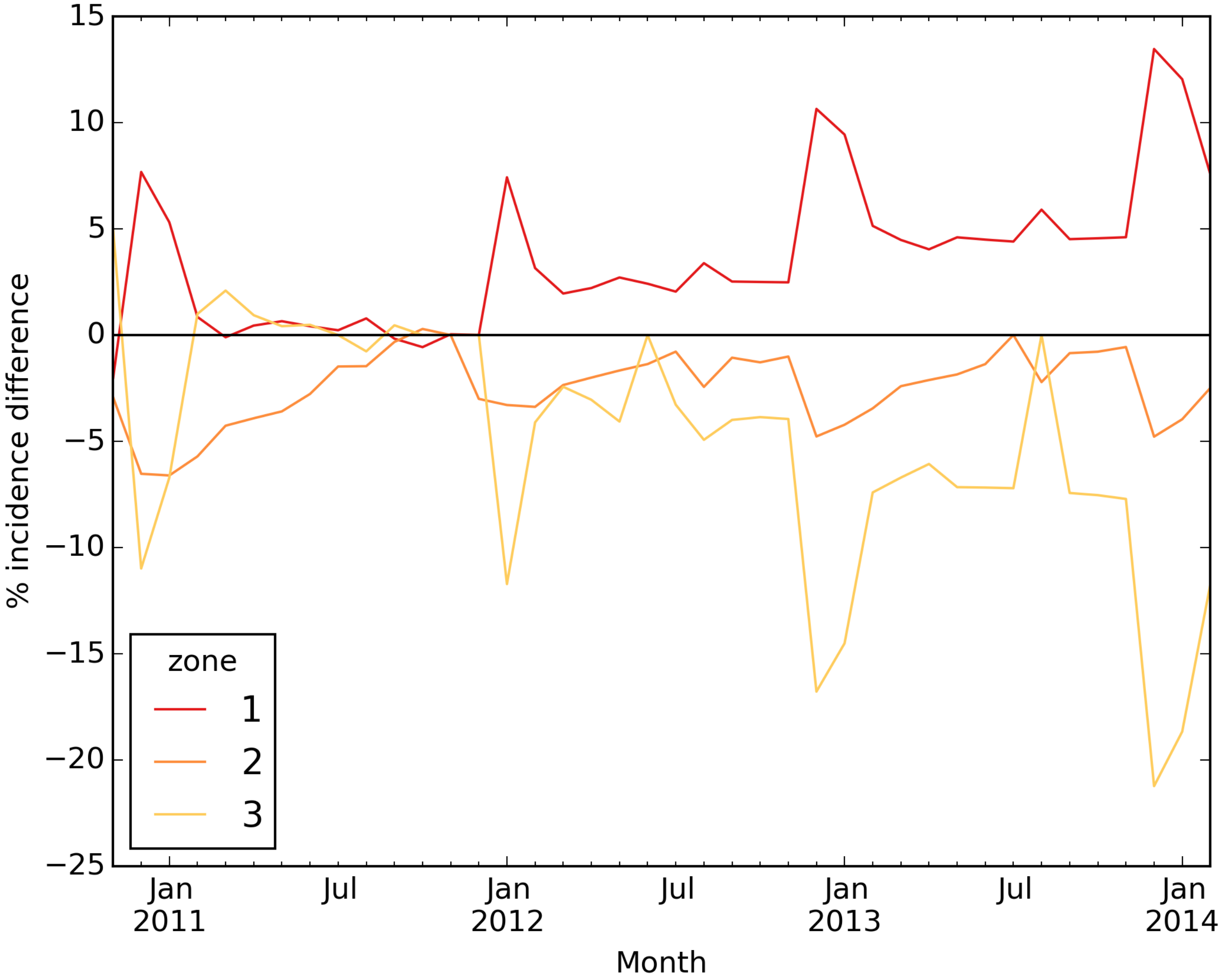

Figure 5: Difference in incidence using static and dynamic denominators by malaria zone.

3.2 Health district level

The insets in Figures 4 and 6 in the main manuscript show predicted population numbers and dynamic incidence respectively, over time for selected health districts. Figure 6 shows predicted population numbers over time for all health district, Figure 7 shows dynamic incidence.

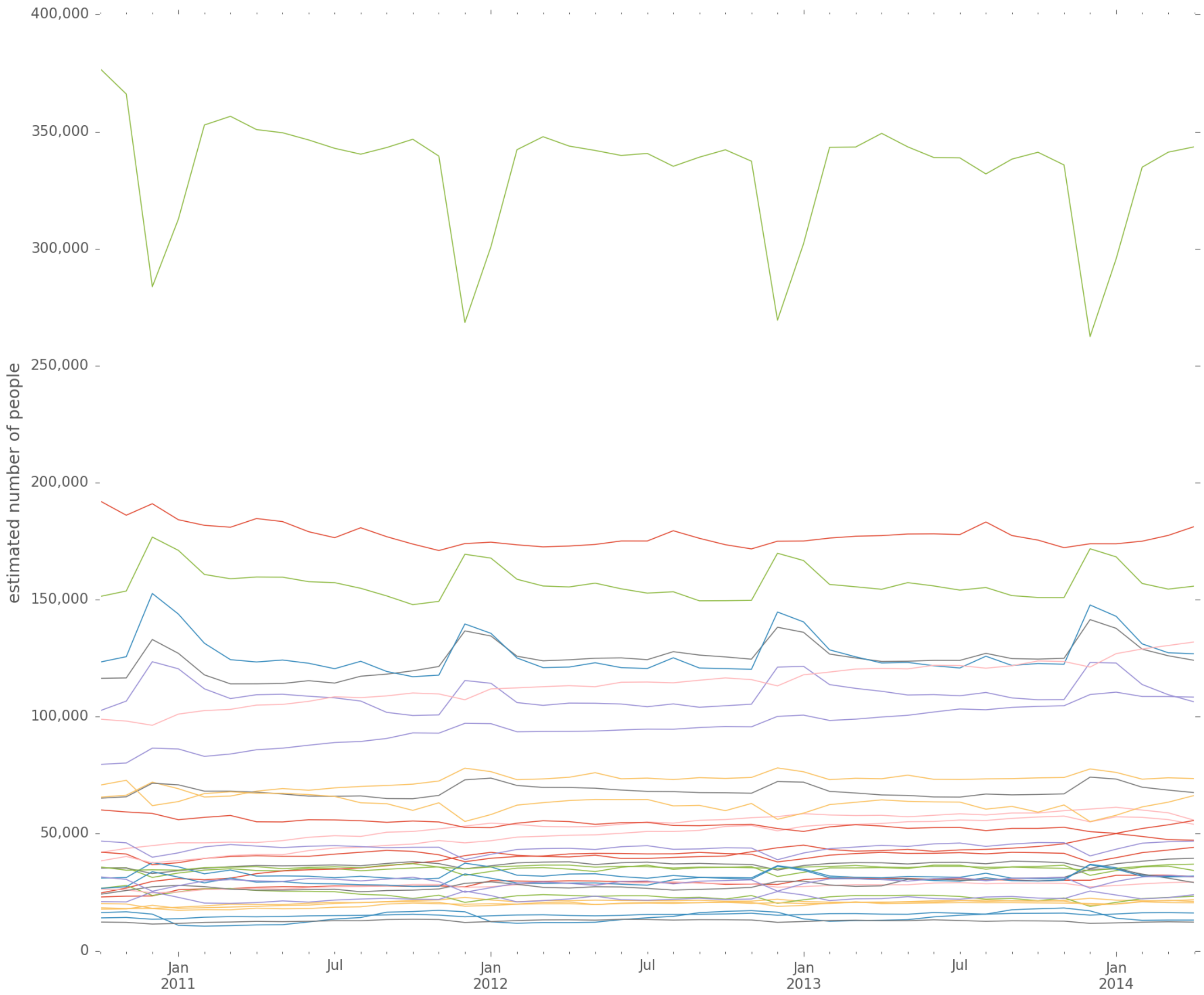

Figure 6: Population predictions for each health district.

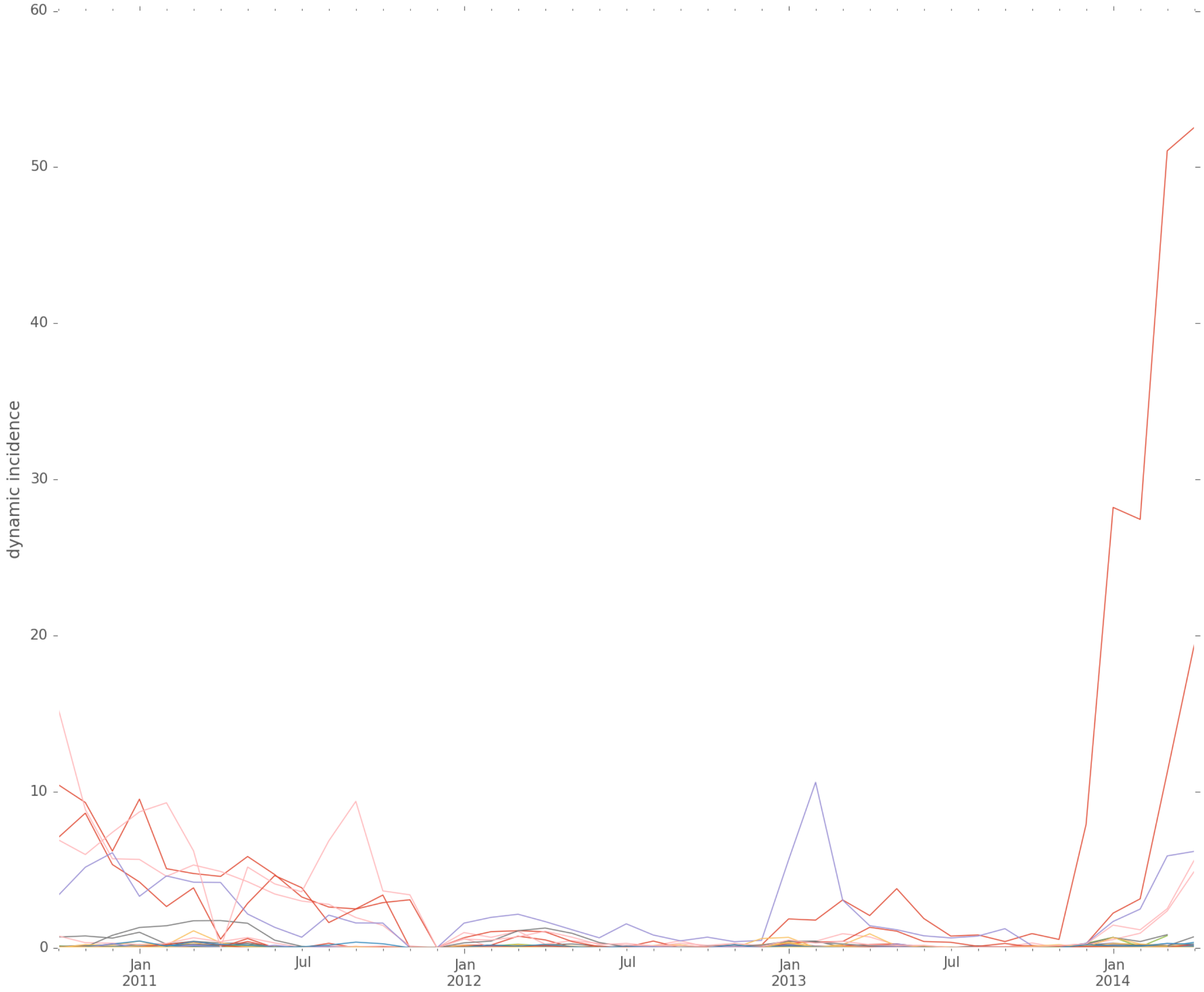

Figure 7: Dynamic incidence over time for each health district.
